# Expression of Concern: Brain–Computer Interface–Based Communication in the Completely Locked-In State

**DOI:** 10.1371/journal.pbio.3000527

**Published:** 2019-10-16

**Authors:** The PLOS Biology Staff

The editors have become aware of an internal investigation by the authors’ institution (Eberhard Karls Universität Tübingen) recommending withdrawal of the manuscript due to alleged breaches of good scientific practice, including selective data selection during data collection. A press release by the authors’ institution regarding its findings (in German) can be found here.

The authors have presented to the editors an unofficial translated version of the institution’s decision (originally in German) and have informed PLOS that they have appealed the decision. The case was being investigated by the German Research Society (Deutsche Forschungsgemeinschaft; DFG), which has just released a press release regarding its investigation on Sept 19th 2019.

The journal will be processing this case according to the guidelines provided by the Committee on Publication Ethics (COPE, https://publicationethics.org). In the meantime, this Expression of Concern should be taken to indicate that the data and conclusions offered in the study may not be reliable. *PLOS Biology* will take further action, as relevant, after consulting with the authors’ institution and the DFG.
